# Modified precordial lead ECG SafOne on electrocardiography recordings

**DOI:** 10.1038/s41598-022-12013-x

**Published:** 2022-05-13

**Authors:** Wan Nishfa Dewi, Safri Safri, Iswadi Hasyim Rosma

**Affiliations:** 1 Department of Medical Surgical Nursing, Faculty of Nursing, Universitas Riau, Pekanbaru-Riau, Indonesia; 2 Department of Electronic Engineering, Faculty of Engineering, Universitas Riau, Pekanbaru-Riau, Indonesia

**Keywords:** Cardiology, Health care

## Abstract

Adaptability in precordial lead placement is one of the sources of electrocardiography inaccuracy. The present experimental study aimed to investigate the modified precordial lead ECG SafOne on electrocardiography recordings. Fourteen subjects were selected using purposive sampling. All the artefacts that emerged in the ECG recording results from the subjects using both the modified precordial lead ECG SafOne and precordial lead standard ECG were measured and identified. Data were analysed using a t test to examine the difference in the artefacts from all ECG recordings. The electrocardiography recordings of males aged 21–25 years using modified precordial lead ECG SafOne showed that out of 168 precordial leads from 14 subjects, two indicated artefact images in lead II (1.19%) and three in lead III (1.79%). The statistical test showed no significant difference in terms of artefacts that emerged in the electrocardiography recording results from both standard ECG and modified precordial lead ECG SafOne (p = 0.096). The modified precordial lead ECG SafOne showed no significant effect on ECG recordings related to artefacts. Additionally, the precordial lead ECG SafOne had no substantial difference in the presence of artefacts compared to the standard ECG. Therefore, ECG SafOne was usable as an ECG precordial lead for electrocardiography recording.

## Introduction

Electrocardiography (ECG or EKG) recording is one of the most important cardiac diagnostic tests because it is used to determine cardiac problems and is noninvasive, inexpensi^1,2^. This diagnostic tool works with an ECG signal recorded from many electrodes attached over the skin to detect and diagnose cardiac disease^3,4^. This method for electrocardiography recording was invented in the 1930s and established in clinical practice in the 1940s and is currently known as a 12-lead electrocardiogram (ECG)^5^. Since then, a wide range of ECG devices have been used in clinical practice: 12-lead ECG, multichannel ECG (MECG), Holter monitoring, implantable loop recorder (ILR), and others^6–8^. An excellent standard is the well-known 12-lead ECG, where wires are connected to electrodes placed on 10 locations on the body^7,9^. These 12-lead ECGs have recently become the topic of an important and interesting investigation around the globe since their introduction into clinical practice^10^.

The rapid development of technology has a significant impact on new technology, including in the health sector. A study conducted by Bond et al. (2016) developed the CardioQuick Patch (CQP) to assist operators in accurately positioning electrodes during 12-lead ECG acquisition^11^. Similar to ECG SafOne, the CQP aimed to improve the accuracy of electrode placement that can be applied in a time that is comparable to single-electrode application. CPQ and ECG SafOne use different designs, materials and procedures. The CQP uses adaptable horizontal placement and rigid vertical placement designs, which still need time to ensure electrode position when used, while ECG SafOne uses a single simple design. Thus, ECG SafOne is easy and practical in terms of finding the V1–V6 placement, and it is time efficient. CQP can be applied to both males and females of all torso sizes and stay on the patient for up to three days. On contrast, ECG SafOne can only be applied to males with two different sizes of device (medium and large) based on the Indonesian male standard size, and it is not designed for long-term use during the procedure.

Tsukada et al. (2019) and Li, Xiong and Li (2020) developed a measurement of ECG signals based on textile material called the wearable measurement of ECG^6,12^. Both studies aimed to investigate the usefulness of textile electrodes for ECG recording using fabric as the material. Electrode textile pads and lead wires were sewn to a textile that tolerated repeated washings and collected ECG signals during recording. The difference between these two tools is in the utility, where Tsukada’s smart garment is usable for continuous and repeated ECG monitoring, not during dynamic trunk movement, while Li’s smart clothing showed good recording performance in the conditions of sleeping and jogging^6,12^.

This diagnostic test (ECG) is important to record the electrical activities of the heart that can be recorded using electrodes attached to the six thoracic bones and four extremity landmarks. However, errors in the placement of precordial leads, misplacement, and interchange of precordial electrodes will cause invalid ECG recordings^13^. Misconnection or misplacement of the electrodes while performing a 12-lead electrocardiogram can lead to various electrocardiographic changes called artefacts. To collect accurate data, the ECG electrodes need to be correctly applied, in terms of both the electrode position and the adequacy of the conductor; otherwise, an inverted record or artefact will result^13^.

Definitions for precision in the electrocardiographic recording were addressed, and calibration methods were established to measure the timing and amplitude of electrical signals from the heart uniformly. Uniformity standardization for limb lead has been acknowledged; however, precordial lead placement is the most recognized source of variability^5^ and can result in several interpretation errors and artefacts. ECG artefacts are normal beats that result not only from the electrical activity of the heart but also from noise interference^3^. In the last two decades, several studies have investigated under what circumstances 12-lead ECG has been modified with a variety of aims^10^. However, there has been much debate about studies on how ECG morphology can change with different lead placements, particularly in healthy subjects.

When performing ECG under normal conditions, ECG electrodes can be attached properly, but this process sometimes takes time. The misplacement of the ECG electrodes can be avoided by reducing the misplacement of precordial electrodes^11^. However, in responding to emergencies, the misplacement of precordial leads may occur. The condition will be exacerbated if a patient experiencing a heart attack is restless and unstable. As a result, ECG results can fail and affect ECG recordings and lead to incorrect interpretation and diagnosis.

These conditions can be anticipated by finding a practical way to determine the position of the placement of the precordial leads quickly and precisely so that they can be used in any condition. However, there is currently no specific device to determine practical electrodes to point to the position of the precordial lead used to perform ECG tests^6,11,12^. Therefore, an ECG precordial lead modification to answer this question is essential. The current study introduces a new tool designed to correct this problem and facilitate proper precordial lead placement. The authors of this study recently proposed a modified precordial lead ECG SafOne for recording electrocardiography. The present study investigated the modified precordial lead ECG SafOne on electrocardiography recordings. To ensure that this modified precordial lead ECG SafOne is appropriate to record ECG, the examination evaluated the different artefacts that emerged between the precordial lead ECG SafOne and the precordial lead ECG standard.

## Results

### Data demography

Table 1 below shows the characteristics of the subjects based on sex, age, body mass index and rhythm.

**Table 1:** Characteristics of subjects based on sex, age, body mass index and rhythm.

Variable	Frequency		Total	
	n	%	N	%
**Sex**				
Male	14	100	100	100
Female	0	0		
**Age (in years)**				
20	2	14.3	100	100
21	2	14.3		
22	4	28.6		
23	4	28.6		
24	1	7.1		
25	1	7.1		
**BMI**				
Low	2	14.3	100	100
Ideal/normal	8	57.2		
Overweight	4	28.6		
**Rhythm**				
Regular	14	100	100	100
Irregular	0	0	0	

All 14 subjects involved in this study were male (100%), and the majority of respondents were aged 22 and 23 years (57.2%). Based on body mass index (BMI), most respondents had ideal BMI, but some had low and overweight BMI.

### ECG artefacts and rhythms

**Figure 1:**
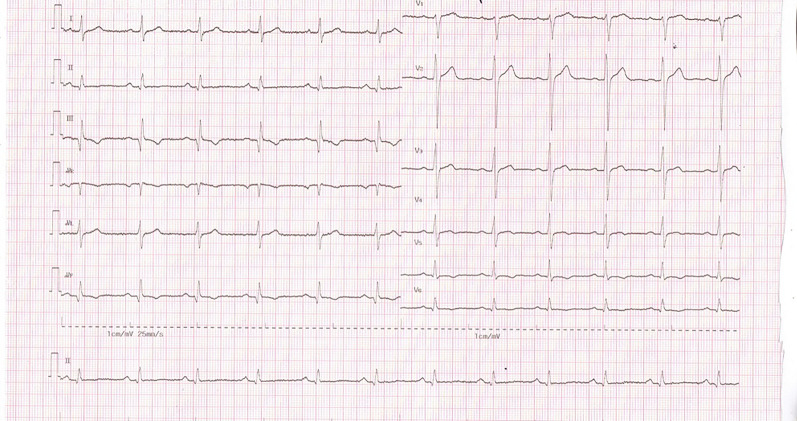
ECG SafOne 12-lead ECG recording.

**Figure 2:**
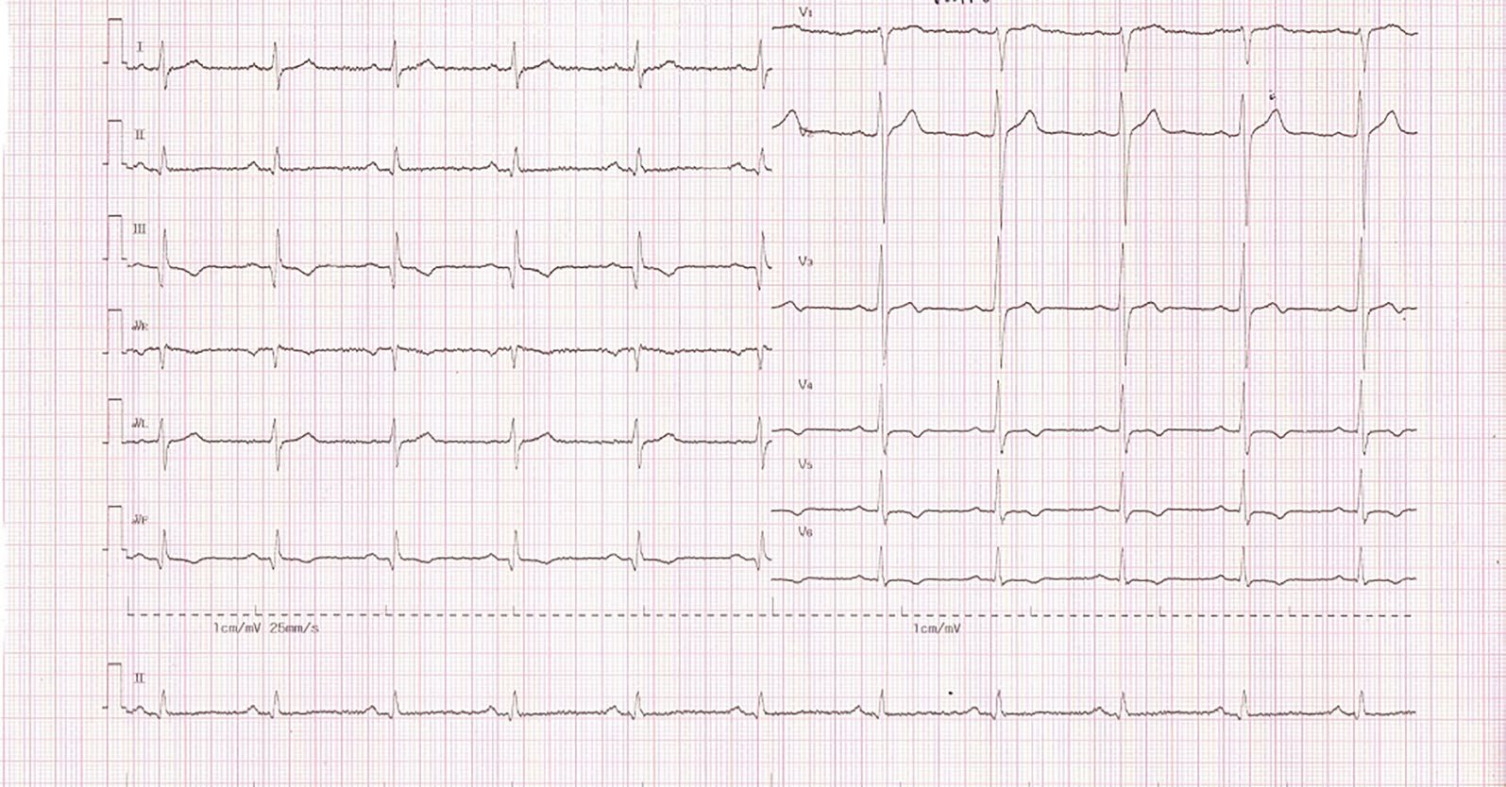
Standard ECG 12-lead ECG recording.

Data collected in this study were 12-lead ECGs recorded on 14 male subjects in sinus rhythm, which discovered no trace of cardiac disorders and were conveyed as being normal limits. In addition, ECG data were normally distributed. The standard 12-lead standard ECG and precordial lead ECG SafOne were recorded at standard ECG paper speed in the supine position for healthy male subjects, as shown in Figs. 1 and 2.

As shown in Figs. 1 and 2, the ECG recordings acquired from both the precordial lead ECG SafOne and the standard ECG depicted proper images, although the presence of artefacts on the ECG recordings of the ECG leads emerged. Table 2 below describes the overview of artefacts collected from all the subjects participating in this study. There were 168 ECG recording leads from 14 subjects, and five of the leads had artefacts in leads II and III. Artefacts in lead II occurred in two subjects (1.19%), and artefacts in lead III occurred in three subjects (1.79%).

**Table 2:** Overview of artefacts on 168 ECG leads of 14 respondents.

ECG morphology	Total lead	n	%
**Artefacts**			
Lead II	168	2	1.19
Lead III		3	1.79

The mean of artefacts on standard ECG was 0.00, indicating no artefacts (standard deviation is 0.000). Meanwhile, on the precordial lead ECG SafOne, the artefact mean was 0.36, with a standard deviation of 0.7945 (p = 0.096). In summary, there was no significant difference between the standard ECG results and the results of the ECG recordings using a modified precordial lead ECG SafOne (see Table 3).

**Table 3:** Artefact differences between standard ECG and precordial lead ECG SafOne.

Variable	Mean	SD	SE	N	P value	
**ECG morphology**						
Standard ECG artefacts	0.00	0.000	0.000	14	0.096	No significant difference
Modified precordial lead ECG artefacts	0.36	0.745	0.199			

## Discussion

In the present study, we tested the effects of a modified precordial lead ECG SafOne on electrocardiography recordings and compared it to a precordial lead standard ECG. During the study, the authors found that the precordial lead ECG SafOne placed on the precordial lead subjects produced profound electrocardiography recordings. Using basic morphological criteria of ECG recording, we were able to identify ECG artefacts due to switched electrodes. We confirmed that the ECG SafOne was very comparable to the standard ECG during the study when conducting ECG recordings in the supine position. However, the artefacts that emerged in the present study for both ECGs might be related to noise interference, skin distortion and skin–electrode reciprocal movement. It has been reported that as the precordial leads are unchanged, no significant changes are found in the ECG waveform^14,15^.

Electrocardiographic artefacts are defined as waveform interference or electrocardiographic alterations in an ECG recording resulting from noise interference or anything that is not caused by the electrical activity generated by the heart^3,5,6^. As a result of artefacts, certain sections of the electrocardiogram, such as the baseline and waveform, can be distorted. In general, artefacts can be classified into two types: nonphysiological and physiological artefacts. Artefacts caused by equipment problems or interference from nearby electrical devices are nonphysiological artefacts, while physiological artefacts are caused by muscle activities or skin interference^3^. This study found that ECG recordings from both standard ECG and the ECG SafOne had artefacts in two leads (leads II and III), which may be classified as physiological artefacts. Therefore, the artefacts that emerged in various and uncertain forms in this study did not interfere with the interpretation of ECG results. There were several possible causes for these artefacts: the position of the electrodes, conductors, skin moisture, and pulling between the two limb electrodes^16–19^.

The electrodes should not be placed in an incorrect position, such as above the ribs, because it can inhibit the flow of electricity from the ECG^18,20^. During this study, the positioning of the right precordial lead electrode placement (V5 and V6) was not precise in terms of its location, and it became loose or released. Even though the electrodes in this initial pattern were not placed on the ribs, the subtle artefacts on the ECG were due to the inaccuracy of the attachment position. An earlier study mentioned that one of the frequent mistakes when performing an ECG is the low placement of the right electrode in the 5th intercostal space, which is usually unnoticed due to its position^11,13^.

Conductors and skin moisture are disturbances that occur when an ECG test is performed. These two factors can affect an ECG test result by imaging artefacts, baseline irregularities, electrical interference, and signal weakness^20,21^. Examples of artefacts that are frequently encountered during an ECG are muscle activities and patient movement. Another form of artefact is an ECG baseline aberration, which can occur in fatigued or heavily breathing patients. In this condition, the ECG baseline will appear wavy, not straight, as in a normal ECG^21^. In this study, the artefacts that occurred were most likely due to conductors, skin distortion and skin moisture. This is because skin moisture can be a conductor and can conduct electricity from the respondent's body to the ECG electrodes. It has been estimated that errors in the placement of electrodes occur in 0.4% to 4% of ECGs performed. Incorrect connections of electrodes during ECG recordings may resemble rhythm or conduction alternations^20^. Bond et al. stated that the reasons for the prevalence of precordial lead misplacement are mostly unknown; however, it is well patterned in clinical practice, and to some extent, misplacement of electrodes can affect the clinician’s interpretation of the ECG^11^. Some studies have revealed that errors in locating the precordial lead can impersonate septal myocardial infarction^23^, and misplacing the V1 and the V2 of the precordial lead could stabilize ST elevation and obscure an anterior STEMI^24^.

Based on artefact differences that emerged in this study between the precordial lead standard ECG and the precordial lead ECG SafOne, there was no significant difference between the results of the two precordial lead ECG electrocardiography recordings, and the results did not alter the interpretations of the electrocardiography recordings. The artefacts found during this study did not occur due to improper electrode placement of the ECG SafOne; rather, they may have emerged as a consequence of ECG machine interference when it was connected to an electrical source. The ECG SafOne was designed to easily and accurately locate the precordial position. The ECG SafOne allows health care professionals to locate the V1–V6 position faster by finding the V1 position, and the other precordial electrodes (V2–V6) will automatically follow in their positions. This user-friendly device does not require significant improvement of cognitive effort. Therefore, users can perform ECG recording faster than standard ECG.

Based on the results of this study, it can be concluded that the precordial lead ECG SafOne can be used for ECG recording, as the number of artefacts was minimal and tolerable. However, further research on this device is required to determine the application of the precordial lead SafOne directly into the field of health services and its use by health care professionals.

## Methods

### Study design

This was an experimental study involving 14 male subjects between 20 and 25 years of age with different body mass indices. Ethical approval for this study was obtained from the Ethical Review Board for Nursing and Health Research Faculty of Nursing Universitas Riau with certificate number 45/UN.19.5.1.8/KEPK.FKp/2020. Therefore, all methods were performed in accordance with the relevant guidelines and regulations. Subjects were purposively recruited from the students of the School of Nursing, and all were medically examined to exclude any form of cardiovascular disease. Any subjects with cardiopulmonary diseases that may alter the ECG morphology were excluded from this study. This study was conducted in the medical nursing laboratory in one of the state universities in Indonesia. Two ECG tests using a precordial lead ECG SafOne and a precordial lead standard ECG were equally measured to collect electrocardiography recordings from all subjects. All electrocardiography recordings from both the ECG SafOne and standard ECG were collected and measured for data analysis. Before commencing the study, all subjects involved and participating in this study were given sufficient and clear information regarding the purpose of the study, their involvement and all related knowledge of the procedures and materials used in the study by providing them with an information sheet and an informed consent sheet to be signed if they volunteered to participate in the study. The research team delivered this session. Finally, all subjects gave informed consent to participate in this study.

### Materials and ECG acquisition

**Figure 3:**
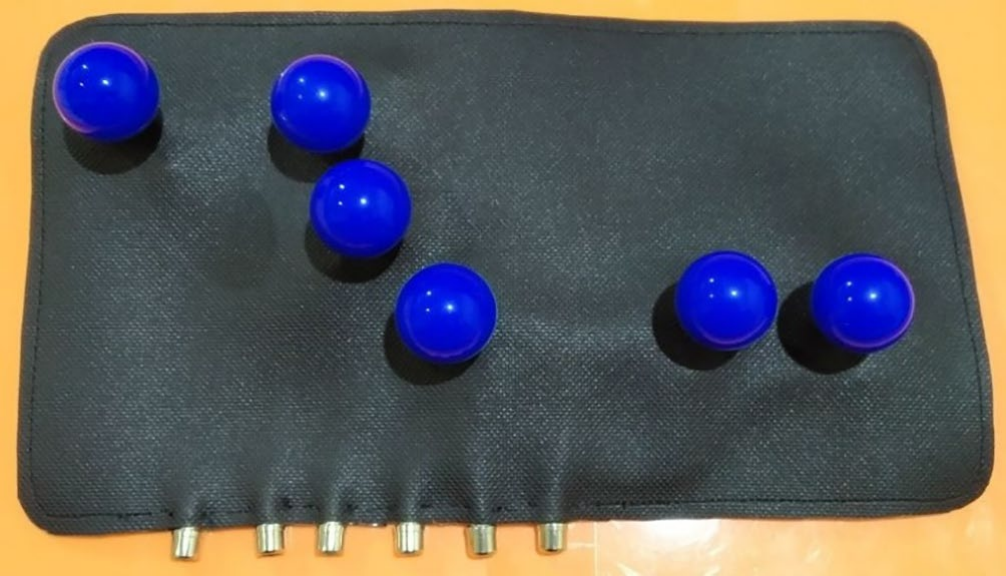
Main view of the ECG SafOne.

**Figure 4:**
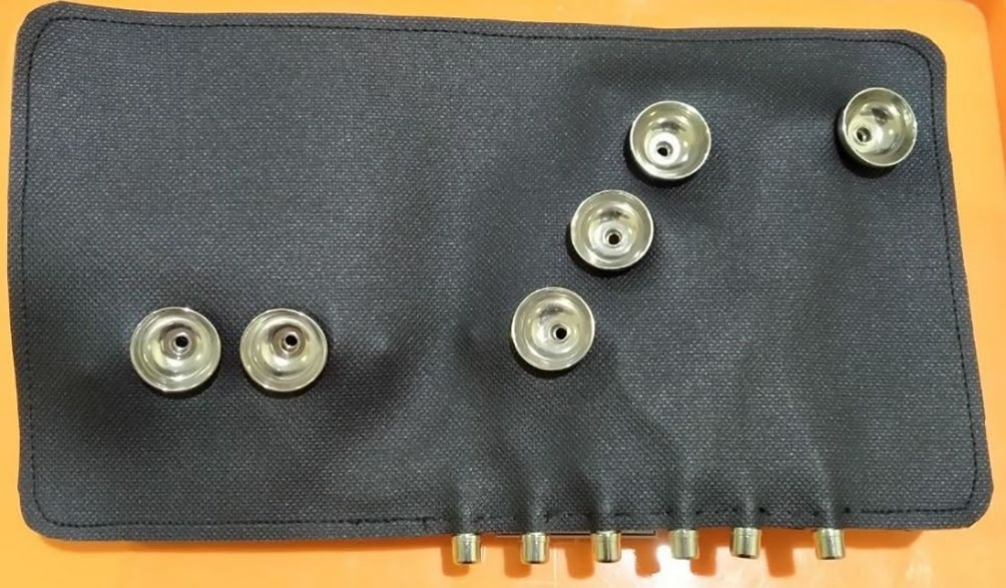
Bottom view of the ECG SafOne.

**Figure 5:**
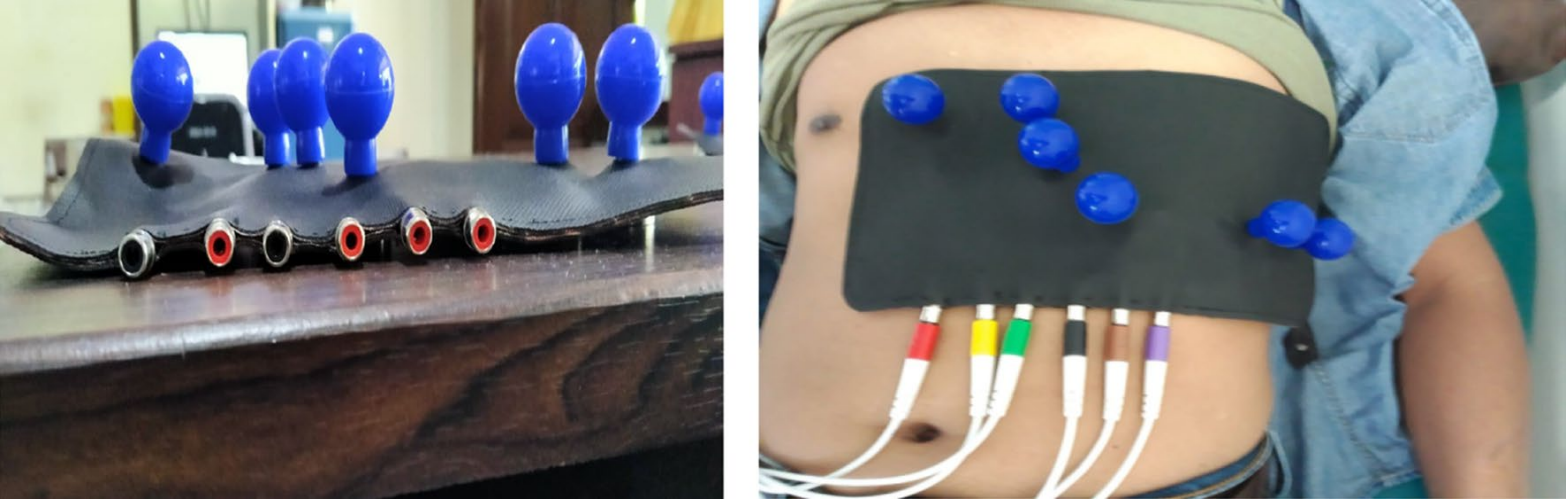
Cable-side view of the SafOne.

The precordial electrode SafOne (Fig. 3) is a smooth and thin synthetic rubber device, sized 10’’–13’’ by 4.6’’–6’’, with six precordial electrodes. Each electrode is equipped with an electrode balloon and a female cable, which are connected directly to the electrode. The bottom part of the ECG SafOne (Fig. 4) is attached to the chest of the patient. This part is the lower part of the electrode (electrode holder). When the electrode balloon above the electrode is pressed, it will produce air suction and will then attach the electrodes to the patient’s chest. A side part of the ECG SafOne (Fig. 5) and a female cable will be connected to the male cable during ECG recording. The cable consists of six pieces, the same as the number of electrodes on the ECG SafOne.

A digital ECG recorder (FUKUDA DENSHI FX-7542, Inc., Japan) operating at 8000 samples per second per channel with a frequency response of 0.05 Hz to 150 Hz was used to acquire ECG data. This ECG recorder is printed on 12-lead ECG paper with a speed from 5 mm/s to 50 mm/s. All ECGs were recorded using the standard 12-lead electrode positions and subsequently by the precordial lead ECG SafOne by repositioning the six precordial leads. ECGs in this study were recorded at standard ECG paper speeds of 25 mm/s and 10 mm/mV.

### Data collection

Collection of data was conducted at the Laboratory of Medical Nursing at the Faculty or Nursing, Universitas Riau. Two research teams experienced in using a standard precordial ECG and the precordial ECG SafOne performed ECG recordings. Consecutive ECGs were recorded using the precordial ECG SafOne and a standard precordial ECG on all healthy subjects involved, with the subjects in the supine position. All ECG recordings, both from standard ECG and the ECG SafOne, were collected for their interpretation.

### Data analysis

All the ECG data were analysed automatically with an ECG measurement and interpretation program on the Fukuda Denshi ECG machine. The presence of artefacts of each 12-lead ECG was recorded and kept for further analysis.

### Statistical analysis

The data were subjected to univariate and bivariate analyses by employing t tests. Data for subject characteristics are presented as frequencies and percentages, means ± standard deviations (SDs), and means ± standard errors (SEs). The t test was used to determine the statistical significance of artefact measurement differences between the precordial lead ECG SafOne and the precordial lead ECG standard. All tests were two-sided, and p < 0.05 was considered statistically significant.

### Study limitations

The limitations for this modified precordial ECG SafOne are that this tool cannot be adjusted to the unique shape of each chest. Furthermore, the ideal distance between each electrode position or point needs correction because the material used to create the modified precordial ECG SafOne is neither clear nor very flexible. The present study results are valid when used for normal conditions; supine sinus rhythm male subjects and female subjects were not included in this study. In addition, this study has not yet studied subjects with known and unknown cardiovascular abnormalities to test whether this modified precordial lead ECG SafOne provides ECG recordings similar to those of standard ECG.

## Conclusion

Electrocardiography artefacts occur frequently and may be readily identified in most cases. They involve external and internal interference, such as poor grounding of the device, interference by noise and devices within the room, chest compression and decompression, and mistaken placement of the precordial leads. This work shows that this user-friendly ECG SafOne is a capable solution to the electrode misplacement problem, particularly for emergency situations. The ECG SafOne was usable as an ECG precordial lead for ECG recording, and it was demonstrated that the ECG SafOne recording results were not significantly different compared to the ECG recording results from standard ECG. Although artefacts still appeared in the precordial lead ECG SafOne recordings, they did not alter the interpretation of the ECG recordings.
